# In the era of whole-brain mapping for the exploration of mental disorders, we need to rethink our methods of rodent model establishment

**DOI:** 10.1038/s41398-022-01887-0

**Published:** 2022-03-28

**Authors:** Chuanjun Zhuo, Hongjun Tian, Jiayue Chen, Qianchen Li, Lei Yang, Xueqin Song, Yong Xu, Qinghua Luo, Weihua Yue, Chunhua Zhou

**Affiliations:** 1 Department of Key Laboratory of Real Time Imaging of Brian Circuits in Psychiatry and Neurology (RTIBNP_Lab), Tianjin Fourth Center Hospital Affiliated to Tianjin Medical University, Tianjin Fourth Center Hospital, 300140 Tianjin, China; 2 Department of Psychiatry, Tianjin Fourth Center Hospital Affiliated to Tianjin Medical University, Tianjin Fourth Center Hospital, Tianjin, 300140 China; 3 Laboratory of Biological of Psychiatry, Tianjin Anding Hospital, Tianjin mental health Center of Tianjin Medical University, Tianjin, 300222 China; 4 Department of Psychiatry, First Affiliated Hospital of Zhengzhou University, Zhengzhou, 450052 China; 5 Department of Psychiatry, First Hospital/First Clinical Medical College of Shanxi Medical University, Taiyuan, Shanxi Province 030000 China; 6 Department of Psychiatry, the First Affiliated Hospital of Chongqing Medical University, Chongqing, 400016 China; 7 PKU-IDG/McGovern Institute for Brain Research, Peking University, Beijing, 100191 China; 8 Department of Pharmacology, the First Hospital of Hebei Medical University, Shijiazhang, Hebei Province 050000 China

**Keywords:** Neuroscience, Schizophrenia

**Dear Editor**,

The declared aim of *Translational Psychiatry* is to bridge the gap between rapidly accumulating neuroscience knowledge and novel treatment development and application by fostering translation to clinical applications, thereby ultimately transforming healthcare and improving health worldwide. Indeed, several papers published in *Translational Psychiatry* have accelerated translations from laboratory to hospital bed. In a review recently published in this journal, Markicevic et al. [1]. emphasized the need to adopt rodent models for whole-brain mapping; they argued that such adoption will be the trend in future translational psychiatric research and will provide more useful reference information for clinical practice, as rodents’ emotional, recognition, and behavioral expression is more similar to that of humans than is that of mice. We strongly agree with this view, but we think that the methods used to establish such models need to be carefully considered.

In past decades, murine models of mania and schizophrenia have been used widely to investigate trajectories of pathological feature alterations, neural activity alterations, and treatment effects in this disease context. Most such models, employed in the past five decades, have been based on the use of ketamine and MK-801 (which induce chemo brain) to induce manic-like and schizophrenic-like symptoms, respectively [2]. During the same period, murine models of depression have been established using methods such as chronic unpredictable mild stress, forced swimming, and tail suspension [3]. With progress in research methods and techniques, this approach of establishing murine models of mania and schizophrenia with drugs, and those of depression with psychological stimuli, has come into question. Its main limitation is that the exogenous infusion of the drugs used in this context causes brain structural and functional alterations; accumulating evidence also indicates that psychological stimuli induce brain structural alterations. With the exception of dementia, mental disorders induced by physical illness, and substance use disorders, most mental disorders (e.g., schizophrenia, major depressive disorder, bipolar disorder, and anxiety disorder) cannot be explored by exogenous drug infusion or psychological stimuli alone.

The etiologies and pathophysiological mechanisms of mental disorders remain incompletely understood, despite great efforts in psychiatric research over the past 100 years. Although several hypotheses regarding bipolar disorder, depression, and schizophrenia—all with supporting evidence—have been proposed in the past 50–60 years, no single hypothesis has been generally accepted or withstood reverse scrutiny. The neurotransmitter-based hypothesis is the oldest and most classic of these hypotheses, and almost all current therapeutic agent use in clinical practice is based on it. The neurodevelopment hypothesis has been popular since the late 20th century and may shape psychiatric research in the next few decades; brain plans put forward by many countries and organizations are based on it. Other hypotheses, such as those involving inflammation, neuroimmunity, vitamin and trace element deficiencies, and intestinal flora, as well as those based on multi-omics research, are being integrated into research in this field [4]. As pointed out by Zhuo et al., however, the fact that “the reverse inference is not tenable” remains a key issue in psychiatric research [5].

The human brain cannot be imitated perfectly; some scholars describe the exploration of the biological markers and etiological and pathophysiological characteristics of mental illness as seeking hope in despair [6]. In this context, the use of rodent models, established with multiple techniques, for the whole-brain mapping of neural activity can provide very useful clues about mental illness, and represents the new era of translational psychiatry. Compared with the mouse brain, the rodent brain more closely resembles the human brain. However, the establishment of an ideal rodent model is a new and important challenge in translational psychiatry. Currently, rodent models mimicking depression in humans depend on the induction of chronic social defeat and stress (social brain) [7]. However, the simultaneous achievement of high degrees of surface, structural, and predictive validity with current animal models of stress is difficult. In addition, these models are not applicable to all animal types, and sex differences exist. Damri et al. [8]. proposed the more advanced approach of using mitochondrial-respiration inhibitors to establish a novel model of bipolar disorder(Gene brain). Bipolar disorder has been associated with mitochondrial dysfunction, including down-regulation of the expression of mitochondria-related genes and proteins and mitochondrial DNA mutations. In addition, most patients with bipolar disorder show symptoms of mitochondrial disorder. Thus, rotenone, a mitochondrial-respiration complex I inhibitor, has been used to induce behaviors associated with mania and depression, accompanied by relevant changes in mitochondrial basic oxygen consumption rate and mitochondrial respiratory protein levels, but this approach cannot currently be applied to rodents. Currently, most rodent models of mania are established with ketamine injection, and those of schizophrenia are established with phencyclidine [9]. Thus, many current approaches to the establishment of rodent models of mental illness follow those used for murine model establishment; no “breakthrough” method has been recognized. Hence, as proposed in *Translational Psychiatry* [8], the exploration of new methods of rodent model establishment is an urgent task required for the acceleration of translational medicine, with the ultimate aim of improving treatment effect for patients with mental illness.

In 2011, Young et al. proposed that the search for a better treatment for mania ideally requires the establishment of fully novel animal models that facilitate the application of comprehensive test batteries to examine multiple aspects of mania, utilization of etiological validity from genetic perspectives, and objective quantification of behavior. This recommendation, and recommendations for practical approaches to the investigation of mania using rodent models [10], have received insufficient attention in the past decade. In future research, we must consider how to avoid the limitations of previous models, improve existing methods, and develop new methods for the establishment of ideal large animal models in translational psychiatry, to fulfill the crucial objective of improving the treatment and life quality of patients with mental disorders.

Some scholars have proposed that the adoption of new techniques, such as the use of injectable flexible electrode film, enables direct testing in human studies, without first using animal models. This film is an extremely soft strip-grid electrode that can be curled easily to form a slender needle, facilitating injection and thereby avoiding a large number of surgical transplants. In one experiment, part of a flexible reticular brain electrode was injected into mouse brains through skull perforations, and part of it was connected to an external computer to control the application of electrical stimulation and monitor neuron activity [11]. This new technology enables the stable long-term monitoring of cell activity at the neuronal level in mouse brains, with little damage to neurons. It improves the biocompatibility of brain grafts and enables more accurate treatment of depression and schizophrenia [11]. However, the computational complexity of human brains is billions of times that of rodent brains, and computing speeds will not be able to accommodate such complexity in the next decade. Despite much progress, such techniques are not currently suitable for the exploration of new methods to improve patient prognoses [5].

Hence, the methods described above cannot overcome the limitations of chemo brain or the difficulty of achieving social brain to provide information aiding the improvement of treatment methods from other perspectives. In the future, new whole-brain imaging technologies for exploration of the mechanisms underlying schizophrenia and mania will be developed through multidisciplinary collaboration. Although many difficulties exist, the work is moving forward.
